# Morphology Study on Inclusion Modifications Using Mg–Ca Treatment in Resulfurized Special Steel

**DOI:** 10.3390/ma12020197

**Published:** 2019-01-09

**Authors:** Ping Shen, Jianxun Fu

**Affiliations:** 1 State Key Laboratory of Advanced Special Steel; Shanghai Key Laboratory of Advanced Ferrometallurgy; School of Materials Science and Engineering, Shanghai University, Shanghai 200444, China; koulvpinlei@126.com; 2 School of Metallurgical and Ecological Engineering, University of Science and Technology Beijing, Beijing 100083, China

**Keywords:** modification, MnS, Al_2_O_3_, inclusion, Mg–Ca treatment

## Abstract

In resulfurized special steel, MnS and Al_2_O_3_ are two main inclusions that deteriorate fatigue life and machinability. It is important that these two inclusions should be well controlled to increase steel quality and usage performance. In the present study, a Mg–Ca treatment was employed to modify the MnS and Al_2_O_3_ inclusions in resulfurized steels to reduce detrimental effects on fatigue life and machinability. In the laboratory study, Ni–Mg alloy was added to 16MnCrS5 and 49MnVS3 steels. Both Al_2_O_3_ and CaO–Al_2_O_3_ were gradually modified to MgO·Al_2_O_3_ and MgO, being surrounded by MnS, that is, a complex inclusion with an oxide core and sulfide outer layer was formed. The amount of the complex inclusion increased with Mg content. In the hot forging experiment, non-Mg treated inclusions were in the morphology of long strip, while those with Mg treatment were seen to be less deformed with spherical morphology of low aspect ratio in which case inclusions had less effect on steel mechanical properties. The Mg–Ca treatment was also applied to the manufacture of resulfurized special steel in steel plants. The scanning electron microscope–energy dispersive spectrometer and statistical results agreed well with those in the laboratory studies.

## 1. Introduction

Resulfurized steels, such as non-quenched and tempered steel (S = ~0.02–0.065% [1]), gear steel (S = ~0.015–0.035% [2]), etc., are widely used in hot forging automotive components of power transmission system. According to the quality requirement of automotive components, the resulfurized special steel should have excellent fatigue life [3] and good machinability, both of which are strongly affected by the inclusions in the steel. Manganese sulfide is the main inclusion in the resulfurized special steel. It has a good plasticity and can be easily deformed into a long stripe after rolling, which causes the anisotropy of steel properties, therefore reducing the fatigue life [4,5], and is also harmful to the machinability of steels [6]. The morphology control of sulfide is the key to the improvement of steel properties. Sulfide with a spherical or spindle morphology is usually beneficial for the mechanical property and machinability of steels [7]. On the other hand, Al_2_O_3_ is another kind of inclusion generated by the Al deoxidation in molten steels. It can become the crack source or promote the crack propagation under alternating load in service, which suppresses the anti-fatigue property [8,9]. In addition, Al_2_O_3_ can cause excessive tool wear in the machining process.

The MnS and Al_2_O_3_ inclusions are two aspects that limit the application of steels. If the detrimental effect of both MnS and Al_2_O_3_ can be eliminated, such as forming a complex inclusion with an oxide core and sulfide outer layer, the deformation of sulfide will be restricted [7], and the oxide will not directly cause the cracks of steels. This will result in the remarkable quality improvement of steels. Based on this idea, Ca treatment and Mg treatment are two manufacturing techniques that have been used in the refining process.

Calcium treatment is usually used in Al-killed steels to modify the Al_2_O_3_ inclusion to liquid calcium aluminates, which can prevent the nozzle clogging [10]. Besides, the calcium aluminates is softer than Al_2_O_3_, and has less of a possibility for the formation of cracks [11]. In the resulfurized steel, Ca is also used to modify the MnS inclusion. Calcium dissolves into MnS and reduces the plasticity of MnS, restricting the deformation of MnS during the rolling process [12]. In the solidification process of steels, sulfide has a strong tendency to precipitate around oxide inclusions, forming complex inclusions. Lind and Holappa [11] investigated the reaction between CaO and Al_2_O_3_ as well as between calcium and Al_2_O_3_ inclusions in steel melts, and put forward the mechanism and kinetics of alumina inclusion modification. Qiao et al. [13] studied the thermodynamics on the modification of inclusions by Ca treatment and stated the control target of Ca content to attain the most ideal effect. Blais et al. [14] studied the effect of different Ca/S ratios on the modification of inclusions and proposed that an optimum Ca/S ratio of 0.70 allowed modification of the mean shape factor of MnS inclusions; the further increase of Ca concentration did not affect the morphology of inclusions. Although, inclusions in steels can be modified by Ca, the modification efficiency is usually not so high, meanwhile the excess addition of Ca may cause the formation of high melting point inclusion CaS [15], leading to the nozzle clogging. In the field of high-end automobiles, the Ca treatment process still cannot meet the requirements of steel property.

Magnesium treatment is another refining process. Compared with Ca treatment, Mg has a stronger affinity for oxygen and sulfur, and the steel shows a better cleanliness after Mg treatment [16]. The Al_2_O_3_ can be modified to MgO·Al_2_O_3_ and MgO, which have a smaller size than that after Ca treatment and distribute dispersedly [17]. The tiny and randomly distributed MgO·Al_2_O_3_ and MgO usually have few influences on steel properties. Additionally, MgO·Al_2_O_3_ and MgO are excellent heterogeneous nucleating agents [18] for the precipitation of MnS. Similar to Ca, Mg can also dissolve into MnS, forming the (Mn,Mg)S solid solution. Zhang et al. [18] reported that MnS preferred to precipitate on the surface of Mg-containing oxide inclusions. The inclusion changed in different orders in the steel with different sulfur contents. Fu et al. [19] studied the modification of inclusions with Mg treatment in 35CrNi3MoV steel, and found that elongated MnS inclusions was replaced by small ellipsoidal MgS·MgO and MgS·MnS·MgO. In the research of Tsunekage and Tsubakino [7], the morphology of inclusions and the mechanical property in the resulfurized steel after Mg treatment were studied. In the steel, (Mn,Mg)S with a fusiform morphology was generated, and the transverse impact properties were remarkablely improved, while the longitudinal impact properties were not affected. The Mg treatment can achieve a better inclusion modification; however, the reaction in the molten steel with the adding of Mg is usually too violent to control in the refining process. Besides, the Mg may also cause the nozzle clogging. Therefore, it is not widely used yet.

Both Mg treatment and Ca treatment can effectively modify the MnS and Al_2_O_3_ inclusions in steels. Nevertheless, the Ca treatment has low modification efficiency, while the Mg treatment may cause the nozzle clogging. Besides, the previous studies mainly focused on the composition change of inclusions, and the morphology change was not well studied. Therefore, Mg–Ca treatment was employed for resulfurized special steel in the present study to achieve a better modification of inclusions without nozzle clogging. The effect of modification on the morphology change of inclusions was systematically studied. The experiments were conducted in both laboratory and industrial conditions. The modification mechanism of inclusions was proposed.

## 2. Laboratory Study

In this section, two kinds of steels, non-quenched and tempered steel 49MnVS3 and gear steel 16MnCrS5, were studied. The chemical composition of the steels is shown in Table 1. The raw materials used for the laboratory study were taken from the center of the continuous casting slabs. During the manufacture of steels, the molten steels were already treated with Ca in the refining process. The Ca content in 16MnCrS5 and 49MnVS3 was 12 ppm and 6 ppm, respectively.

### 2.1. Sample Preparation

**Figure 1:**
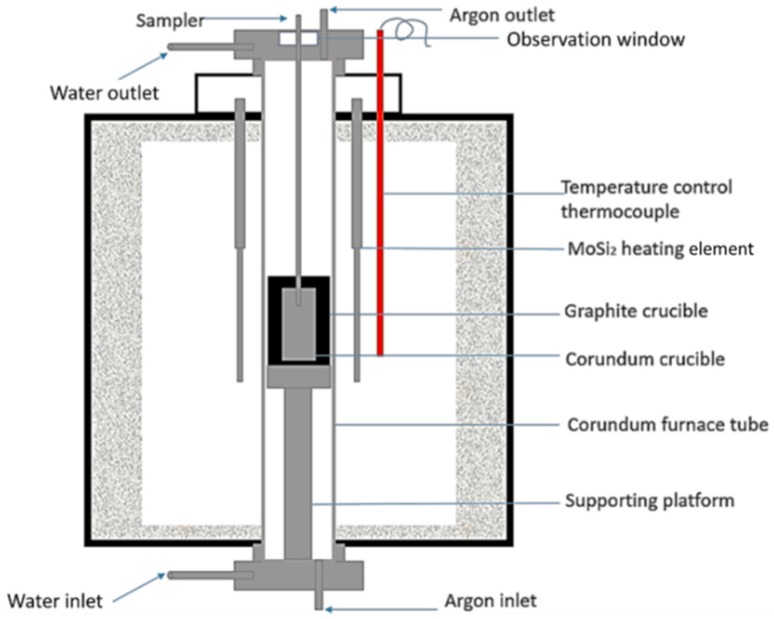
Schematic of the experimental apparatus.

In each experiment, an approximate 500–600 g steel sample was put in a corundum crucible with inner diameter of 53 mm and height of 120 mm, and protected by an outer graphite crucible. Then specimens were placed in the constant temperature zone of a vertical tube resistance furnace. The experimental apparatus is shown schematically in Figure 1. Argon gas was purged into the reaction tube at a flow rate of 1 L/min. Both the graphite crucible and Ar gas were aimed to prevent the oxidation of samples. The temperature was raised to 1000 °C at a heating rate of 8 °C/min and then to 1600 °C at a heating rate of 4 °C/min. The temperature was held at 1600 °C for 20 min, ensuring the complete melting of the steel. Then Ni–Mg alloy wrapped by iron foil was added into the molten steel. The composition of Ni–Mg alloy is listed in Table 1. Five minutes later, the molten steel was stirred with a molybdenum stick. Another 10 min was maintained to ensure the composition homogeneity of the molten steel. Thereafter, the steel was cooled down at a cooling rate of 4 °C/min. The temperature difference between the steel sample inside the corundum tube and the thermocouple outside the corundum tube is approximately 50 °C. Therefore, the temperature of the steel sample was 1550 °C when the furnace showed 1600 °C.

After the smelting trial, the Mg content in each ingot was measured by inductively coupled plasma-optical emission spectrometry (ICP-OES, tested by NCS Testing Technology CO., LTD., Beijing, China), the result is shown in Table 2. For comparison, the re-melted steel sample without adding Ni–Mg alloy is also listed in Table 2. The Mg content in the original steel sample is less than 5 ppm, according to the following analysis of inclusions by EDS (Energy Dispersive Spectroscopy, scanning electron microscope, Phenom, Eindhoven, The Netherlands), few Mg can be detected. Thus, the Mg content is roughly considered to be 0 ppm in the following discussions. Since the original steel sample was already treated by Ca, the re-melted sample is a Ca treated sample, while that with the addition of Ni–Mg alloy is an Mg–Ca treated sample.

In the center of each ingot, a steel sample with a detecting area of 10 mm × 10 mm was machined. After polishing, the steel samples were ultrasonic cleaned in ethyl alcohol. These samples were prepared for SEM-EDS analysis. Except for the observations via two-dimensional morphology of inclusions on the polished surface of steel samples, the non-aqueous solution electrolytic etching method was also used to obtain the stereoscopic structure of the inclusions for the three-dimension morphology observation.

### 2.2. Analysis of Inclusions

**Figure 2:**
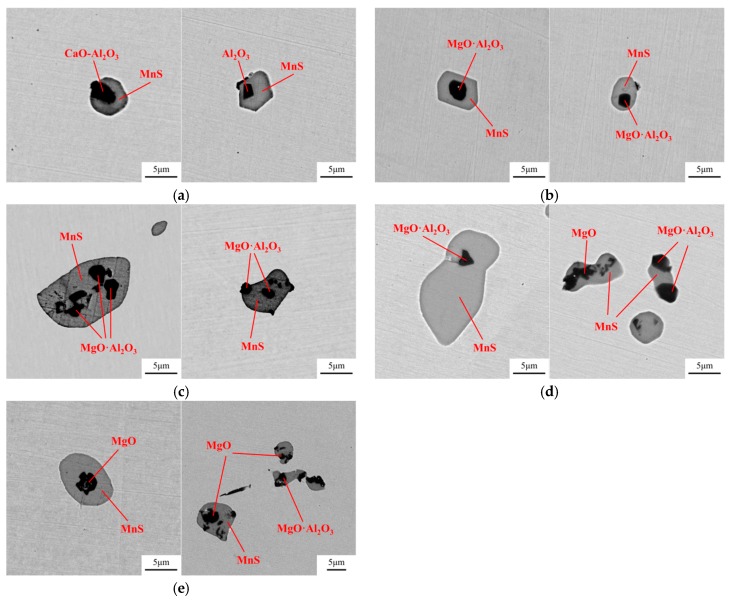
Compositions of the complex inclusions in 16MnCrS5 steels with (**a**) 0 ppm Mg; (**b**) 8 ppm Mg; (**c**) 15 ppm Mg; (**d**) 19 ppm Mg; (**e**) 35 ppm Mg.

The inclusions in both 49MnVS3 and 16MnCrS5 steels were detected by SEM-EDS. With the increase of Mg content in the steel, the variation of inclusions for both steels was similar. For the convenience of discussion, the SEM-EDS result of only 16MnCrS5 steel is discussed. In the steels with different Mg contents, both single MnS and complex inclusions existed. Figure 2 shows the compositions of complex inclusions. In the re-melted sample, the oxide core was mainly composed of CaO–Al_2_O_3_ or Al_2_O_3_. With adding of Mg, the oxide core transformed into MgO·Al_2_O_3_. With the further increase of Mg content, MgO started to generate and became the core of complex inclusion. In the outer MnS layer, there was a small amount of Ca and/or Mg.

**Figure 3:**
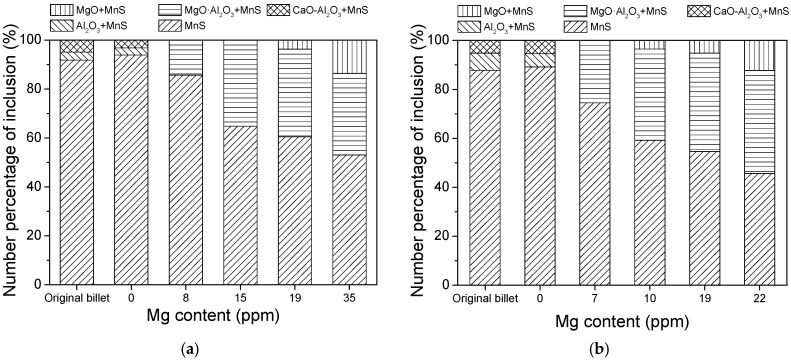
Number percentage of different kinds of inclusions in the steels: (**a**) 16MnCrS5; (**b**) 49MnVS3.

In order to distinguish the ratio of different kinds of inclusions, more than 100 inclusions were detected with EDS in each steel sample. The main inclusion in the current specimens was MnS, of which some were pure single-phase MnS, while some were MnS with oxide cores. Figure 3a shows the counted result for the gear steel 16MnCrS5. The composition and number percentage of each kind of inclusion did not show a big difference between the original billet and the re-melted sample, indicating that the re-melting process barely affected the composition of inclusions. The main inclusions in the original billet and re-melted steel were single MnS, MnS with an Al_2_O_3_ core, and MnS with a CaO–Al_2_O_3_ core. With the increase of Mg content in the steel, the Al_2_O_3_ and CaO–Al_2_O_3_ gradually changed to MgO·Al_2_O_3_ and MgO, which were usually distributed with smaller size [16]. Manganese sulfide did not usually exist in molten steel at 1550 °C. During the solidification of steels, MnS precipitated from the molten steel in the form of heterogeneous nucleation. The oxide inclusions in the steel became the heterogeneous nucleation points for MnS. The more oxide cores, the larger number of complex inclusions. Thus, the amount of single MnS continuously decreased from 93.9 to 53.1% with the increase of Mg content from 0 ppm to 35 ppm. The complex inclusions usually had a higher hardness than MnS and showed less deformation during the rolling process.

In the 49MnVS3 steel, except for the MnS and oxide inclusion, there was also another kind of inclusion, that was (Ti,V)(C,N). However, only single MnS and MnS with an oxide core were counted, the result is shown in Figure 3b. The inclusions in the re-melted sample was also similar to that in the original billet. In both samples, there was a small amount of MnS with an Al_2_O_3_ or CaO–Al_2_O_3_ core. After adding 7 ppm Mg, the Al_2_O_3_, and CaO–Al_2_O_3_ disappeared. Meanwhile, the spinel phase MgO·Al_2_O_3_ was generated. When the Mg content increased, the variation of inclusion type and number percentage of each kind of inclusion were similar to that in 16MnCrS5 steel, where the amount of complex inclusion was obviously increased.

**Figure 4:**
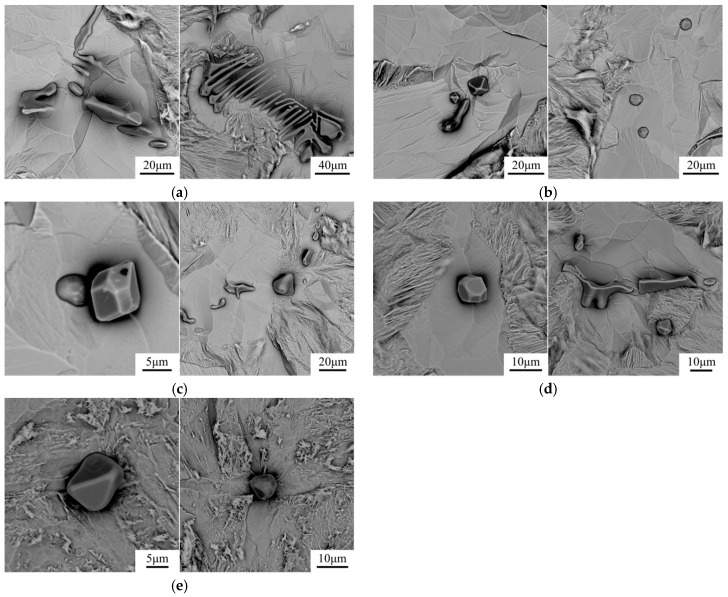
Three-dimension morphology of inclusions in 16MnCrS5 steels with (**a**) 0 ppm Mg; (**b**) 8 ppm Mg; (**c**) 15 ppm Mg; (**d**) 19 ppm Mg; (**e**) 35 ppm Mg.

After electrolytic etching of steel samples in non-aqueous solution, the three-dimension morphology of inclusions was observed with SEM. Figure 4 shows the SEM images of inclusions in steels with different Mg contents. In the re-melted sample, most of the inclusions were type II inclusions with a dendritic shape [20]. With the increase of Mg content, this kind of inclusion gradually transformed to type III or type I inclusion with an angular or spherical shape. Therefore, the modification of inclusions by Mg had a prominent effect on the morphology of inclusions.

### 2.3. Hot Forging Experiment

In order to analyze the deformability of inclusions after Mg–Ca treatment in the hot rolling process, the hot forging experiment was conducted for 16MnCrS5 steel to simulate the hot rolling process. Steel ingots were forged in the radial direction, therefore the MnS inclusion was elongated to the transverse-axial direction, which is similar to the deformation of MnS in the rolling direction for the steel slab. Steel samples with 25 mm in diameter and 35 mm in height were heated to 900 °C at a heating rate of 5 °C/min. Then the steel samples were forged at this temperature for 5 min to reach the same deformation. Subsequently, the forged samples were cooled down to room temperature. Then the steel samples along the transverse-axis direction were prepared for the optical microscope (Carl Zeiss, Oberkochen, Germany) observation and inclusions were counted with Image Pro Plus 6.0 (Media Cybernetics, Rockville, MD, USA).

**Figure 5:**
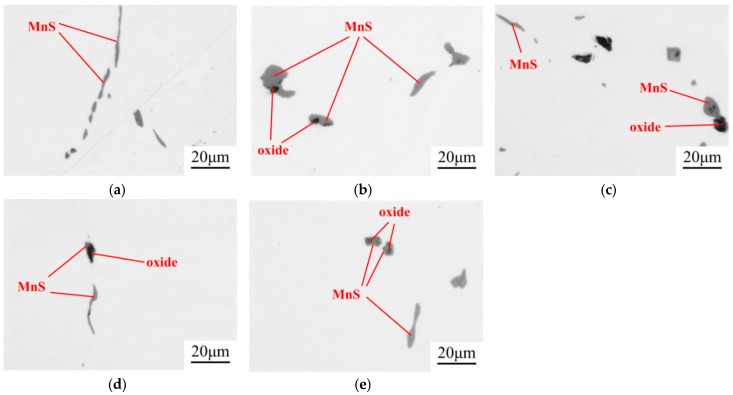
Inclusions in the 16MnCrS5 steel after forging (**a**) 0 ppm Mg; (**b**) 8 ppm Mg; (**c**) 15ppm Mg; (**d**) 19 ppm Mg; (**e**) 35 ppm Mg.

**Figure 6:**
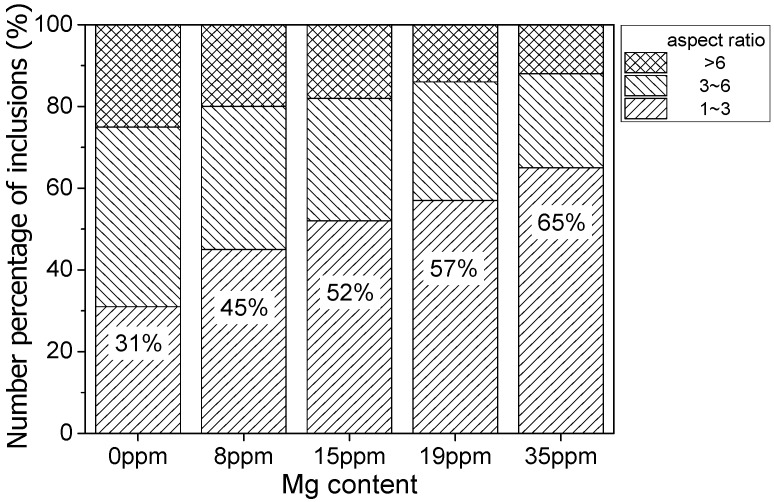
Number percentage of inclusions in 16MnCrS5 steel in different aspect ratio ranges.

The inclusions after the hot forging experiment were captured by an optical microscope, the images are shown in Figure 5. The MnS inclusion without an oxide core was more likely to deform into a long strip. This kind of inclusion existed in all samples. However, the number of long-strip MnS decreased with the increase of Mg content. On the other hand, the complex inclusions with an oxide core showed less deformation. The aspect ratio of inclusions was counted with Image Pro Plus 6.0. It was roughly divided into three regions, as shown in Figure 6. The inclusions with an aspect ratio of ~1–3 was usually considered to be harmless to the steel properties [21]. The number percentage of inclusions with this morphology was increased from 31 to 65% when the Mg content increased from 0 ppm to 35 ppm. Obviously, the addition of Mg had a significant influence on preventing the deformation of MnS.

In the present study, the plasticity of the MnS was reduced by the following two mechanisms:

(1) Calcium or magnesium dissolved into MnS, forming (Mn,Ca)S, (Mn,Mg)S or (Mn,Ca,Mg)S solid solution, which had a lower plasticity than pure MnS [5,12]. During the rolling process, the inclusion showed less deformation performance [7] and kept a low aspect ratio.
(2) Compared with the single MnS inclusion, the oxide inclusion had a higher strength and hardness [22]. The oxide restricted the deformation of MnS inclusion during the rolling process when it became the core of MnS. Magnesium modified the Al_2_O_3_ inclusions, forming large quantity and dispersive distributed MgO·Al_2_O_3_ spinel inclusion or MgO inclusion with a relatively small size [18,23]. It was reported that MgO·Al_2_O_3_ and MgO inclusion had a much weaker tendency to aggregate than Al_2_O_3_ inclusions [17,18,24]. During the solidification of steels, MnS took the tiny spinel inclusion or MgO inclusion as the heterogeneous nucleation point, forming the complex inclusion. The increase of Mg content caused the proportion increase of this kind of inclusion, which was beneficial to restrict more inclusions to deform into a long strip.

## 3. Mechanism of the Modification of Inclusions

### 3.1. Modification of Oxide and MnS Inclusions

Both Mg and Ca are in group two of the periodic table. There are some similarities in the modification of inclusions. Both MnS and Al_2_O_3_ inclusions can be modified by Ca and Mg. At high temperature, the oxide inclusions already existed in the molten steel, while MnS precipitated in the solidification process [25]. Thus, when Ca or Mg was added into the molten steel, the oxide inclusions were first modified. During the solidification of the steel, MnS precipitated around oxide inclusions, forming complex inclusions with inner oxide core and outer MnS layer. Meanwhile, the MnS might also be modified to CaS, MgS or solid solutions (Mn,Ca)S, (Mn,Mg)S, (Mn,Ca,Mg)S. The detailed modification mechanisms of inclusions are discussed as follows.

**Figure 7:**
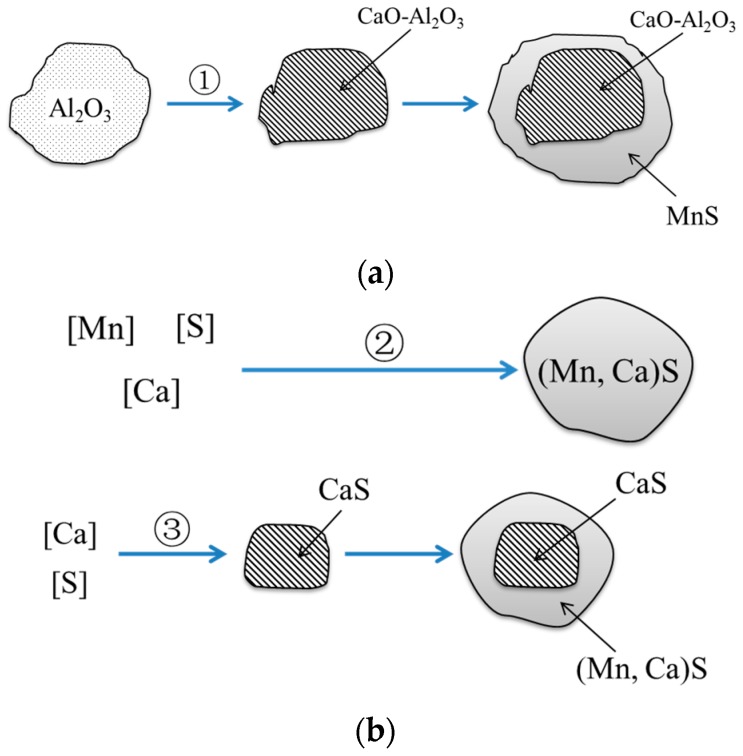
Mechanism of the modification of inclusions by Ca treatment: (**a**) modification of oxide inclusions; (**b**) modification of sulfide inclusions.

When the molten steel was treated with Ca, Al_2_O_3_ transformed to CaO–Al_2_O_3_ with different stoichiometric ratios [9,26,27]. During the solidification of steels, the segregation of Mn and S caused the precipitation of MnS, which wrapped CaO–Al_2_O_3_, as shown in Figure 7a. Meanwhile, a certain amount of Ca might dissolve into MnS, forming the (Mn,Ca)S solid solution. When the Ca content in the steel was high enough, CaS could generate in molten steel, and then wrapped by (Mn,Ca)S in the solidification process. The modification of MnS is shown in Figure 7b.

**Figure 8:**
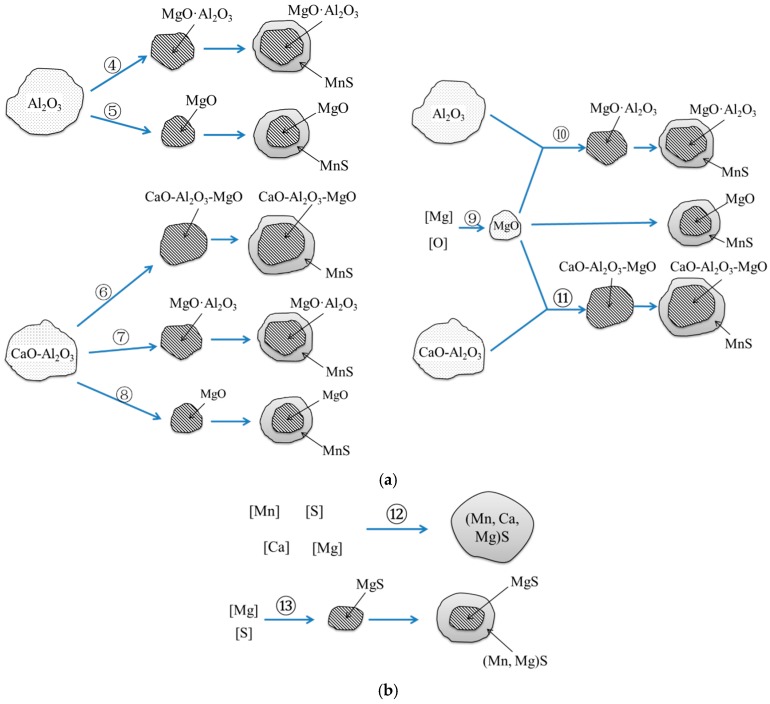
Mechanism of the modification of inclusions by Mg–Ca treatment. (**a**) modification of oxide inclusions; (**b**) modification of sulfide inclusions.

In the Mg–Ca treated steel, Al_2_O_3_ was already modified by Ca in the raw material. After re-melting of the steel, the main oxide inclusions in the molten steel were CaO–Al_2_O_3_, as well as a certain amount of un-modified Al_2_O_3_. After the addition of Mg, the Al_2_O_3_ could change to MgO·Al_2_O_3_ and MgO, which were excellent heterogeneous nucleation point, inducing the precipitation of MnS on the surface of MgO·Al_2_O_3_ and MgO. The modification of Al_2_O_3_ by Mg is shown in Figure 8a. On the other hand, when modified by Mg, the CaO in CaO–Al_2_O_3_ inclusion might be replaced by MgO, forming CaO–Al_2_O_3_–MgO or MgO·Al_2_O_3_. When more Mg was added, the CaO–Al_2_O_3_ might transform to MgO. In addition, when Mg was added into the molten steel, part of Mg became the deoxidizer and reacted with the dissolved oxygen, forming MgO [28]. Then, the MgO reacted with Al_2_O_3_ and CaO·Al_2_O_3_. When the MnS was modified, the formation mechanism of (Mn,Ca,Mg)S, (Mn,Mg)S, and MgS was similar to that modified by Ca. The modification schematic of inclusions is shown in Figure 8b. In the present study, both Ca and Mg content were not high, thus, CaS and MgS were not observed. The reaction of each modification process is listed in Table 3.

### 3.2. Transformation of Inclusions

(1) Effect of Mg content

**Figure 9:**
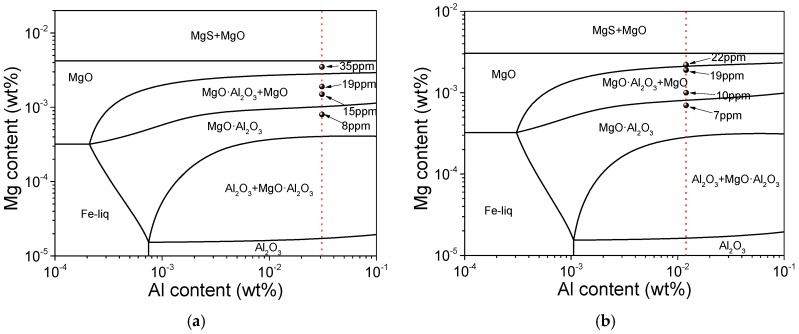
Equilibrium phase diagram of Mg treatment for (**a**) 16MnCrS5 steel; (**b**) 49MnVS3 steel.

According to the composition shown in Table 1, the equilibrium phase diagrams of Al content vs. Mg content at 1550 °C were calculated by FactSage 7.2 using “FactPS”, “FToxid”, “FTmisc” databases, as shown in Figure 9. Since the calcium aluminate almost disappeared after adding a small amount of Mg, for the convenience of analysis, the Ca was not considered in the calculation. The phase diagrams for 16MnCrS5 and 49MnVS3 were similar. A small amount of Mg could lead to the formation of spinel phase MgO·Al_2_O_3_. The further increase of Mg resulted in the formation of MgO. In both steels, there was a Mg content range that MgO and MgO·Al_2_O_3_ could exist in the molten steel simultaneously. When the Mg content was high enough, MgS might also generate. However, the highest Mg content in the laboratory experiment was still lower than the critical condition for the formation of MgS. The EDS analysis for the inclusions proved this.

(2) Effect of time

**Figure 10:**
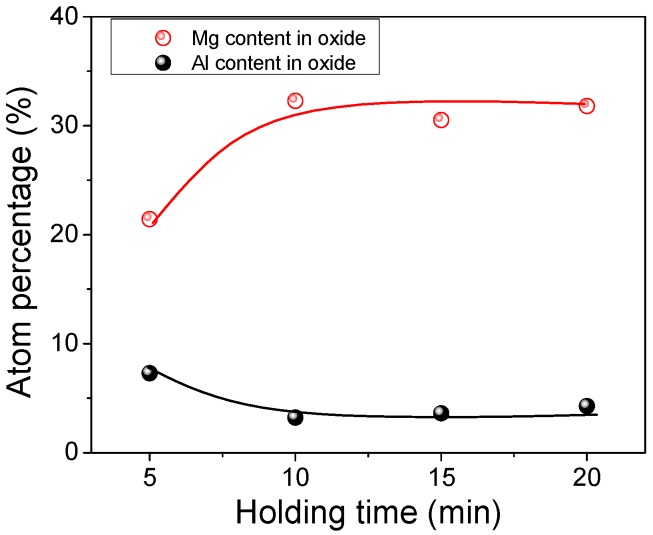
Effect of holding time on the composition of inclusions.

In the present study, another experiment considering the holding time was conducted to analyze the effect of time on the transformation of inclusions. Four samples were taken from the molten steel at 5 min, 10 min, 15 min, and 20 min, respectively, after stirring, and quenched in the water immediately. The samples were cut and polished, preparing for the SEM-EDS analysis. The Mg and Al content in the oxide cores were detected and counted. Figure 10 shows the average atom percentage of Mg and Al in the oxide cores. When the holding time increased from 5 min to 10 min, the Mg content in the oxide core was increased, while the Al content was decreased. The further increase of holding time had little effect on the composition of oxides. Thus, the 10 min selected for the holding time after stirring could ensure the uniformity of the composition.

## 4. Industrial Production

**Figure 11:**
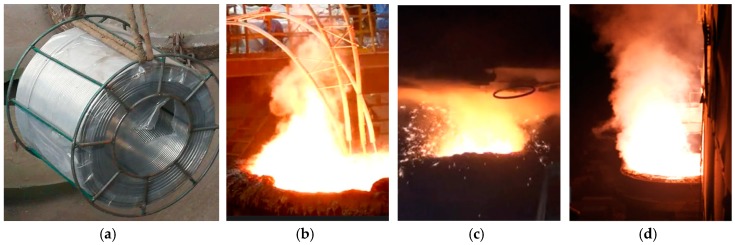
Mg treatment process in a ladle in the industrial experiment. (**a**) Mg-containing core-spun yarn; (**b**) manufacture of 49MnVS3; (**c**) manufacture of 20CrMnTiSH3; (**d**) manufacture of 20CrMnTiH.

According to the laboratory study, the Mg–Ca modification technique was applied in the manufacture of resulfurized special steel in steel plants. Figure 11 shows the photos of the application of Mg–Ca modification in the manufacture of non-quenched and tempered steel 49MnVS3, gear steel 20CrMnTiSH3 and 20CrMnTiH. The production process of each kind of steel is listed as follows:

49MnVS3: EAF→LF→VD→CC

20CrMnTiSH3: BOF→LF→RH→CC

20CrMnTiH: BOF→LF→RH→CC

where EAF is electric arc furnace, LF is ladle furnace, VD is vacuum degassing, CC is continuous casting, BOF is basic oxygen furnace, RH is Ruhstahl–Hausen vacuum degassing process.

In the following paragraphs, the analysis of the industrial experiment of non-quenched and tempered steel 49MnVS3 will be discussed. Table 4 shows the composition control range of the steel.

Calcium and magnesium were added into the ladle in the form of core-spun yarn after VD refining respectively. Magnesium is an active metal and has a high saturated vapor pressure. During the feeding process, Mg could easily evaporate, the reaction and splashing of molten steel were relatively violent than Ca treatment. By adjusting the composition of the core-spun yarn and improving the feeding process, these two issues were controlled to an acceptable level. Steel samples with Ca and Mg–Ca treatment were taken from the hot rolled steel. All samples were cut and polished, preparing for the following analysis. The Ca and Mg content in the Mg–Ca treated steel was 8 ppm and 5 ppm, respectively.

**Figure 12:**
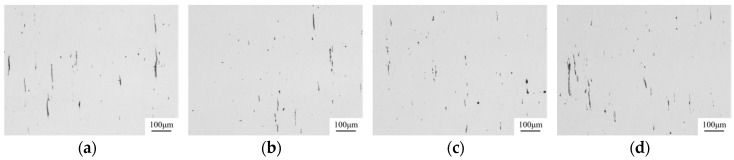
The inclusions in the 49MnVS3 steel after Ca treatment: (**a**) image 1; (**b**) image 2; (**c**) image 3; (**d**) image 4.

**Figure 13:**
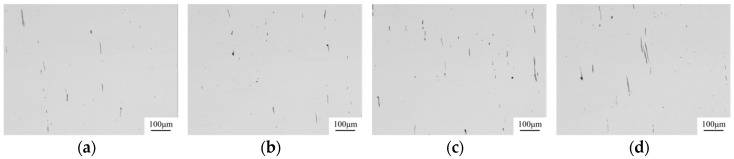
The inclusions in the 49MnVS3 steel after Mg-Ca treatment: (**a**) image 1; (**b**) image 2; **(c**) image 3; (**d**) image 4.

Figure 12 and Figure 13 show the inclusions in the 49MnVS3 steel after Ca treatment and Mg–Ca treatment captured by the optical microscope. The MnS inclusions in the Ca treated steel mainly showed strip morphology. While in the steel treated with Mg–Ca, MnS tended to keep a spherical or spindle-shaped morphology. Besides, the average equivalent diameter of inclusions decreased from 6.5 μm to 5.3 μm. In order to evaluate the effect of Mg–Ca treatment on the inclusions in the steels, the inclusions in both Ca treated steel and Mg–Ca treated steel were determined with the standard ISO 4967: 1998. Table 5 shows the result of the assessment of sulfide inclusions in both steels. Compared with the Ca treatment, the assessment of sulfide inclusions after Mg–Ca treatment was much better. The index number was approximately 0.5 lower than that of Ca treatment for both the average fine series and thick series, while for the worst fine series and thick series, the index number was 1.0 lower. Obviously, the inclusions in the steel with Mg–Ca treatment achieved a big improvement.

**Figure 14:**
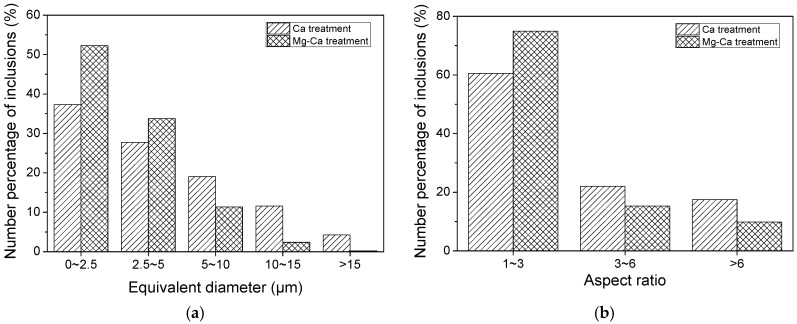
Number percentage of inclusions in 49MnVS3 steel in (**a**) different diameter ranges; (**b**) different aspect ratio ranges.

The inclusions in both steels were counted using Image Pro Plus 6.0 software. The statistical results are shown in Figure 14. A remarkable number increase of small inclusions could be observed, and more inclusions were in the aspect ratio range of ~1–3. Taking all the inclusions into consideration, the average statistical results of inclusions are shown in Table 6. Compared with the Ca treatment, the diameter, area fraction, and aspect ratio achieved a big decrease, while the number density increased after Mg–Ca treatment. It meant that the additional Mg treatment could modify the inclusions to dispersive distributed tiny inclusions, providing more heterogeneous nucleation points for the precipitation of MnS. Besides, the decrease of area fraction of inclusions indicated that the steel was purified by the Mg treatment, as was the same in references [16,19]. Therefore, the Mg treatment had a significant effect on the modification of inclusions, preventing the deformation of inclusions, which was consistent with the result in the laboratory study.

**Figure 15:**
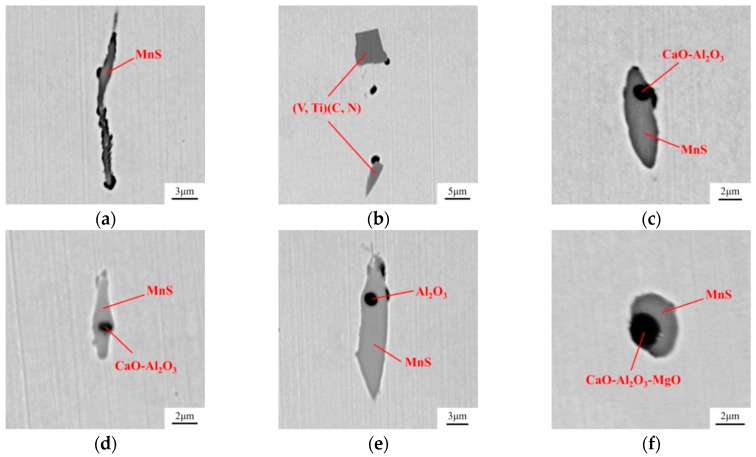
Compositions obtained from the EDS results of inclusions in the steel after Ca treatment. (**a**) MnS; (**b**) (V,Ti)(C,N); (**c**) CaO–Al_2_O_3_ + MnS; (**d**) CaO–Al_2_O_3_ + MnS; (**e**) Al_2_O_3_ + MnS; (**f**) CaO–Al_2_O_3_–MgO + MnS.

**Figure 16:**
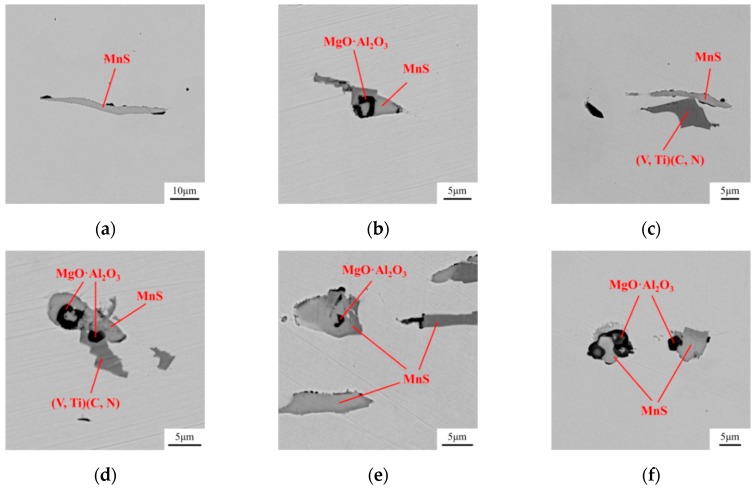
Compositions obtained from the EDS results of inclusions in the steel after Mg–Ca treatment. (**a**) MnS; (**b**) MgO·Al_2_O_3_ + MnS; (**c**) (V,Ti)(C,N) + MnS; (**d**) MgO·Al_2_O_3_ + MnS + (V,Ti)(C,N); (**e**) MgO·Al_2_O_3_ + MnS; (**f**) MgO·Al_2_O_3_ + MnS

**Figure 17:**
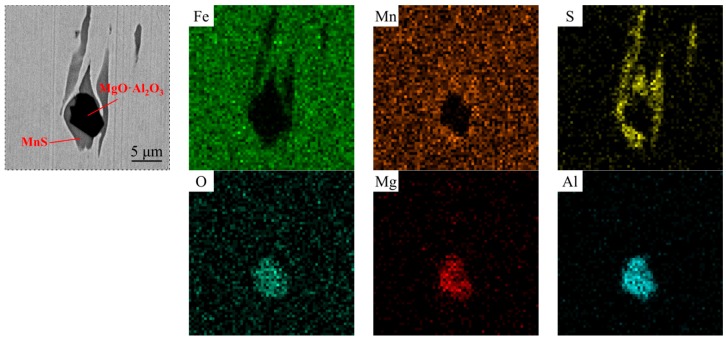
Mapping result of the complex inclusion after Mg–Ca treatment.

The steel samples were then analyzed by SEM-EDS. Figure 15 and Figure 16 show the inclusions in steels after the two treatments. In the Ca treated steel, part of Al_2_O_3_ was modified to CaO–Al_2_O_3_ inclusion, while the rest remained unchanged. In the Mg–Ca treated steel, few Al_2_O_3_ and CaO–Al_2_O_3_ could be observed, most oxide inclusions had been transformed to MgO·Al_2_O_3_. The mapping result of a complex inclusion after Mg–Ca treatment is shown in Figure 17. As mentioned above, the Mg content in the steel was 5 ppm. The detection result was consistent with the previous thermodynamic analysis. This amount of Mg was already high enough to modify Al_2_O_3_.

In both steels, there was a certain amount of single-phase MnS inclusion, which tended to become a long strip. The fewer number percentage of this kind of inclusion, the better morphology of the inclusions.

**Figure 18:**
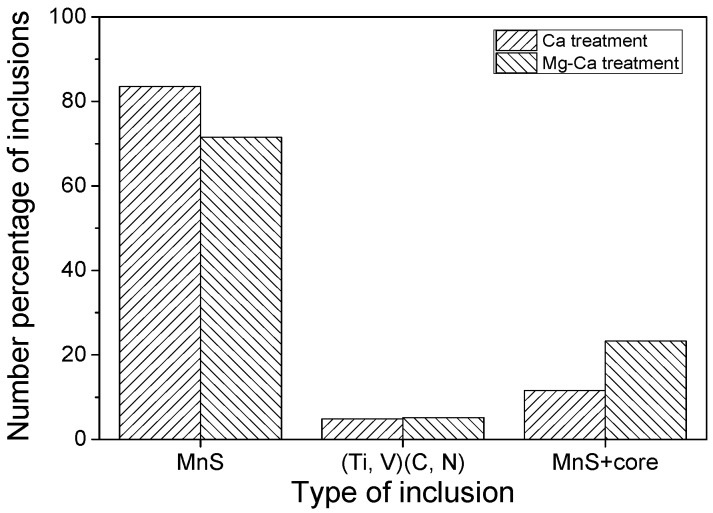
Number percentage of different kinds of inclusions in 49MnVS3 steel.

The inclusions in the steels could be roughly divided into three categories, i.e., single MnS inclusion, MnS inclusion with an oxide core, and (Ti,V)(C,N) inclusion. The number percentage of each kind of inclusion is shown in Figure 18. The number percentage of MnS inclusion with an oxide core increased from 11.6 to 23.3% with the additional Mg treatment, while that of single MnS decreased. Thus, the variation of these two kinds of inclusions proved that the Mg–Ca treatment delivered better inclusions overall compared to the simple Ca treatment. In addition, the (Ti,V)(C,N) inclusion seems to be not affected by the process.

## 5. Conclusions

(1) In the laboratory study, Ni–Mg alloy was added into the Ca treated steel. With the increase of Mg content, the Al_2_O_3_ and CaO–Al_2_O_3_ gradually transformed to MgO·Al_2_O_3_ and MgO. Besides, part of Mg dissolved into MnS, and a solid solution was generated. The oxide inclusion was wrapped by sulfide in the solidification process, forming a complex inclusion with an oxide core and sulfide outer layer. With the increase of Mg content, the number percentage of complex inclusions increased, while the ratio of single MnS decreased. In addition, more inclusions transformed from type II inclusion to type III or type I inclusion.
(2) In the hot forging experiment of 16MnCrS5 steel, the MnS inclusion without an oxide core was deformed into a long strip. While the complex inclusions showed less deformation and kept the morphology with low aspect ratio. With the increase of Mg content, the number percentage of inclusions with a small aspect ratio range increased. The addition of Mg had a significant influence on preventing the deformation of MnS inclusion.
(3) The Mg–Ca modification process employed in the manufacture of non-quenched and tempered steel 49MnVS3 indicated that the extra Mg treatment resulted in the improvement of the assessment of sulfide inclusions compared with that with only Ca treatment. More inclusions had a small size and less deformation of inclusions occurred after hot rolling. The composition and ratio of different kinds of inclusions were in agreement with that in the laboratory study.

## Figures and Tables

**Table 1:** The chemical composition of the samples (wt %).

Composition	Fe	C	Si	Mn	P	S	Al_t_	Cr	V	Ti	Ni	Mg	Ca	O
16MnCrS5	balance	0.16	0.16	1.18	0.0065	0.0278	0.031	1.023	-	-	-	<0.0005	0.0012	0.0019
49MnVS3	balance	0.47	0.35	0.92	0.013	0.047	0.012	0.2	0.1	0.025	-	<0.0005	0.0006	0.0015
Ni–Mg alloy	0.85	0.56	0.11	-	-	-	-	-	-	-	74.19	24.29	-	-

**Table 2:** Mg content in each steel sample (ppm).

Sample No.	1	2	3	4	5
16MnCrS5	<5	8	15	19	35
49MnVS3	<5	7	10	19	22

**Table 3:** Reaction of each modification process.

No.	Reaction	Reference
1	Al_2_O_3_ + [Ca] → CaO–Al_2_O_3_ + [Al]	[29]
2	[Mn] + [S] + [Ca] → (Mn,Ca)S	-
3	[S] + [Ca] = CaS	[9,29,30,31]
4	4Al_2_O_3_ + 3 [Mg] = 3MgO·Al_2_O_3_ + 2 [Al]	[9,32]
5	Al_2_O_3_ + 3 [Mg] = 3MgO + 2 [Al]	[32,33]
6	CaO–Al_2_O_3_ + [Mg] → CaO–Al_2_O_3_–MgO + [Ca]	-
7	CaO–Al_2_O_3_ + [Mg] → MgO·Al_2_O_3_ + [Ca]	[29]
8	CaO–Al_2_O_3_ + [Mg] → MgO + [Ca] + [Al]	-
9	[Mg] + [O] = MgO	[19,32]
10	Al_2_O_3_ + MgO = MgO·Al_2_O_3_	[28,32,34,35]
11	CaO–Al_2_O_3_ + MgO → CaO–Al_2_O_3_–MgO	-
12	[Mn] + [S] + [Ca] + [Mg] → (Mn,Ca,Mg)S	-
13	[Mg] + [S] = MgS	[18]

**Table 4:** Composition control range of 49MnVS3 steel (wt %).

Composition	C	Si	Mn	P	S	Cr	V	Ni	Ti	Al	N
Lower limit	0.46	0.25	0.85	--	0.04	0.15	0.09	--	0.015	--	0.008
Upper limit	0.49	0.4	0.95	0.025	0.06	0.3	0.12	0.2	0.03	0.02	0.02
Target	0.47	0.35	0.9	≤0.020	0.05	0.2	0.1	≤0.20	0.025	0.01	0.015

**Table 5:** Assessment of sulfide inclusions.

Process	Average		Worst	
	Fine Series	Thick Series	Fine Series	Thick Series
Ca treatment	2.0	1.5	3.0	2.5
Mg–Ca treatment	1.5	1.0	2.0	1.5

**Table 6:** Average statistics result of inclusions.

Process	Diameter (μm)	Number Density (mm^−2^)	Area Fraction (%)	Aspect Ratio
Ca treatment	5.17	179	0.52	3.63
Mg–Ca treatment	3.19	263	0.25	2.73
